# Pulsed-field ablation for repeat procedures after failed prior thermal ablation for atrial fibrillation

**DOI:** 10.1016/j.hroo.2024.03.012

**Published:** 2024-04-04

**Authors:** Jens Maurhofer, Hildegard Tanner, Thomas Kueffer, Antonio Madaffari, Gregor Thalmann, Nikola Kozhuharov, Oskar Galuszka, Helge Servatius, Andreas Haeberlin, Fabian Noti, Laurent Roten, Tobias Reichlin

**Affiliations:** ∗ Department of Cardiology, Inselspital, Bern University Hospital, University of Bern, Bern, Switzerland; † Sitem Center for Translational Medicine and Biomedical Entrepreneurship, University of Bern, Bern, Switzerland

**Keywords:** Pulmonary vein isolation, Atrial fibrillation, Pulsed-field ablation, Repeat procedure, Radiofrequency ablation, Cryoballoon ablation

## Abstract

**Background:**

Pulsed-field ablation (PFA) is a novel nonthermal ablation technology. Its potential value for repeat procedures after unsuccessful thermal ablation for atrial fibrillation has not been assessed.

**Objective:**

The purpose of this study was to summarize our initial experience with patients undergoing repeat procedures using PFA.

**Methods:**

Consecutive patients with arrhythmia recurrences after a prior thermal ablation undergoing a repeat procedure using a multipolar PFA catheter from May 2021 and December 2022 were included. After 3-dimensional electroanatomic mapping, reconnected pulmonary veins (PVs) were reisolated and veins with only ostial isolation wither ablated to widen antral PV isolation. Posterior wall ablation was performed if all PVs were durably isolated or in case of low-voltage areas on the posterior wall at the discretion of the operator. Patients underwent follow-up with 7-day Holter electrocardiography after 3, 6, and 12 months.

**Results:**

A total of 186 patients undergoing a repeat procedure using PFA were included. The median number of previous ablations was 1 (range 1–6). The prior ablation modality was radiofrequency in 129 patients (69.4%), cryoballoon in 51 (27.4%), and epicardial ablation in 6 (3.2%). At the beginning of the procedure, 258 of 744 PVs (35%) showed reconnections. Additional antral ablations were applied in 236 of 486 still isolated veins (49%). Posterior wall ablation was added in 125 patients (67%). Major complications occurred in 1 patient (transient ischemic attack 0.5%). Freedom from arrhythmia recurrence in Kaplan-Meier-analysis was 78% after 6 months and 54% after 12 months.

**Conclusion:**

PFA is a versatile and safe option for repeat procedures after failed prior thermal ablation.

## Graphical abstract

**Figure:**
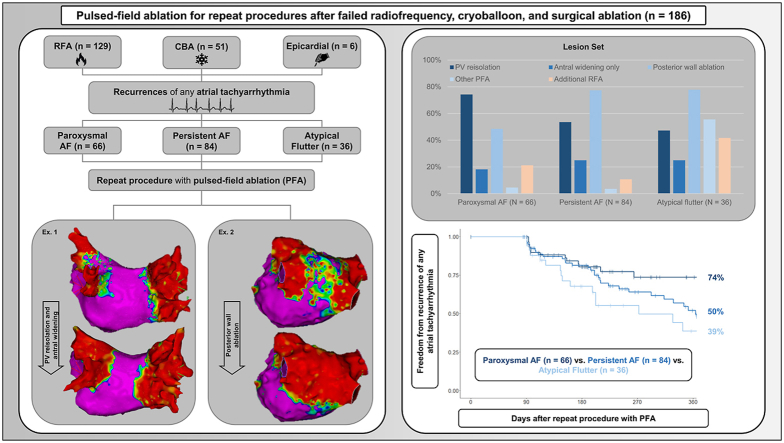

**Key Findings**

▪ In patients with recurrence of atrial tachyarrhythmia after prior thermal ablation, pulsed-field ablation for repeat procedures is safe and versatile.
▪ Pulsed-field ablation in combination with 3-dimensional electroanatomic mapping for repeat procedures is efficient with relatively short procedural times.
▪ Further investigation is needed to gain a better understanding of the role of pulsed-field ablation for repeat procedures, especially in comparison to radiofrequency ablation.

## Introduction

Pulmonary vein isolation (PVI) is a safe, effective, and well-established method for the management of patients with symptomatic atrial fibrillation (AF).^1^, ^2^, ^3^, ^4^ Despite constant optimization of thermal ablation technologies and standardization of ablation protocols, the recurrence rate of atrial tachyarrhythmia (ATa) after PVI remains high.^4^, ^5^, ^6^, ^7^, ^8^, ^9^ A major reason for recurrence after PVI is pulmonary vein (PV) reconnection.^10^, ^11^, ^12^ A repeat procedure for reisolation of reconnected veins is a valid option for many patients with recurrences of ATa after PVI.

In 2021, the first catheter using pulsed-field ablation (PFA) for PVI was introduced to the European market.^13^, ^14^, ^15^ PFA involves the delivery of high-frequency electric pulses to the heart tissue, resulting in the creation of localized areas of myocardial cell death.^16^, ^17^, ^18^, ^19^, ^20^ The clinical use of PFA devices for index PVIs showed a favorable profile in terms of safety and efficacy.^14^^,^^21^^,^^22^ Although most of the clinical PFA experience to date has been obtained from index PVIs, the use of PFA devices might be similarly appealing for repeat procedures because (1) reconnected veins can be reisolated; (2) the level of isolation can be extended more antral for veins with only ostial isolation; and (3) additional left atrial (LA) substrate ablation can be performed if indicated.^23^ Recently, an *in vivo* study of swine reported a high efficiency for repeat ablation with PFA following prior radiofrequency ablation (RFA).^24^ However, the in-human use of PFA for repeat procedures after failed thermal ablation with RFA and cryoballoon ablation (CBA) has not yet been studied.

The aim of this observational study was to investigate the use, safety, and efficacy of a multipolar PFA catheter for repeat procedures after failed thermal AF ablation.

## Methods

### Study population

**Figure 1:**
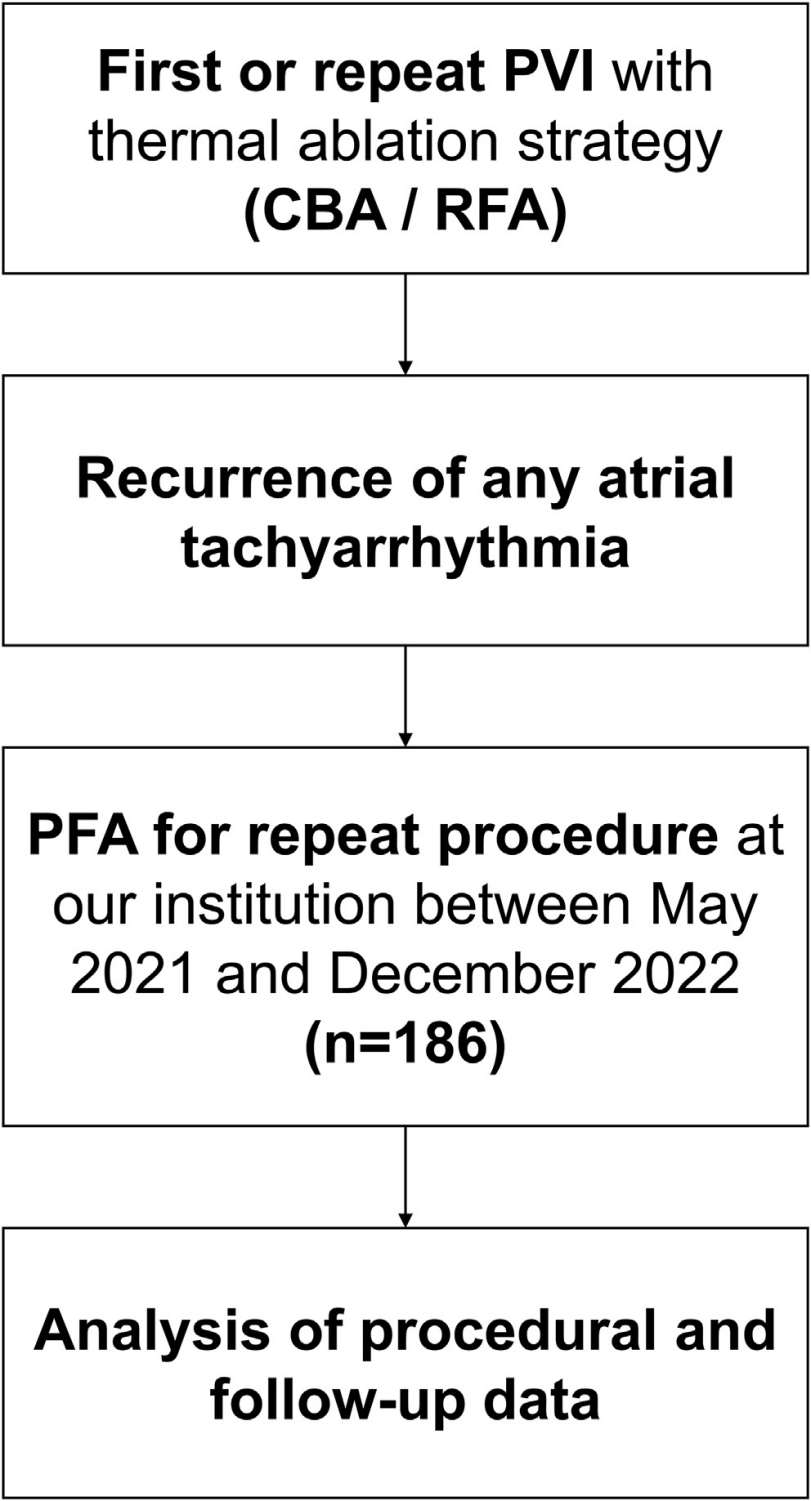
Study flowchart. CBA = cryoballoon ablation; PFA = pulsed-field ablation; PVI = pulmonary vein isolation; RFA = radiofrequency ablation.

In this prospective observational study, consecutive patients with recurrent ATa after thermal AF ablation (either RFA, CBA, or epicardial surgical RFA/cryoablation) and undergoing a repeat procedure using a novel bipolar pentaspline PFA device (Farapulse Inc., Menlo Park, CA) at the Inselspital, Bern University Hospital (Bern, Switzerland) between May 2021 and December 2022 were included. Figure 1 shows a study flowchart for illustration and further information.

Informed consent was obtained from all participants. The registry was approved by the local ethics committee, and the study was carried out in accordance with the principles of the Declaration of Helsinki.

### Protocol for PFA for repeat procedure

Patients underwent transesophageal echocardiography or cardiac computed tomography before the procedure to evaluate LA anatomy and exclude intracardiac thrombi. Deep conscious sedation protocol with midazolam, fentanyl, and propofol was used, with general anesthesia reserved for high-risk patients.^25^ LA access was obtained by fluoroscopy-guided transseptal puncture either using a standard transseptal sheath, followed by an exchange to the 13F Faradrive sheath, or through a direct puncture using the 13F sheath, depending on the physician’s preference.^26^ Heparin was administered to maintain an activated clotting time >350 seconds throughout the procedure.

After successful LA access, a detailed high-density map was acquired using a 3-dimensional (3D) electroanatomic mapping (EAM) system (CARTO3, Biosense Webster, Irvine, CA) and dedicated mapping catheter (PentaRay/OctaRay, Biosense Webster). The 3D EAM model was overlaid on preacquired computed tomographic scans. After evaluation of scar tissue pattern and reconnection status of PVs, the Farawave PFA catheter was inserted via the 13F steerable Faradrive sheath (Farapulse Inc.) into the LA. A detailed description of the PFA platform has been provided previously.^13^ A straight-tip 0.035-inch wire (Amplatzer extra-stiff, Cook Group, Bloomington, IN) or a J-tip 0.035-inch wire (InQwire, Merit Medical, South Jordan, UT) was used to cannulate the PVs. Tissue contact of the PFA catheter was assessed by fluoroscopic imaging.

In case of PV reconnections, reisolation using PFA was performed with at least 8 applications per PV as previously described.^27^ In case of only distal PV isolation, additional PFA applications were performed to widen the isolation to a more antral level. If all veins were already isolated on an antral level and/or if low-voltage areas were found on the posterior wall or previous LA flutter was known, a posterior wall ablation was added with the same PFA device in flower pose at the discretion of the operator. Although this currently is an off-label use of this device, the first experience of this approach has shown favorable results.^14^^,^^28^

Whereas radiofrequency was mostly used for ablation of targets outside the PVs and the posterior wall, PFA was also used in highly selected cases and at the discretion of the operator for isolation of the superior vena cava, for right atrial posterior wall ablation, and/or to complete LA linear ablation at the septum or the posterior mitral isthmus. In case of unsuccessful bidirectional block after PFA, additional RFA (SmartTouch SF, Biosense Webster) was used to reach a durable block in these locations.

**Figure 2:**
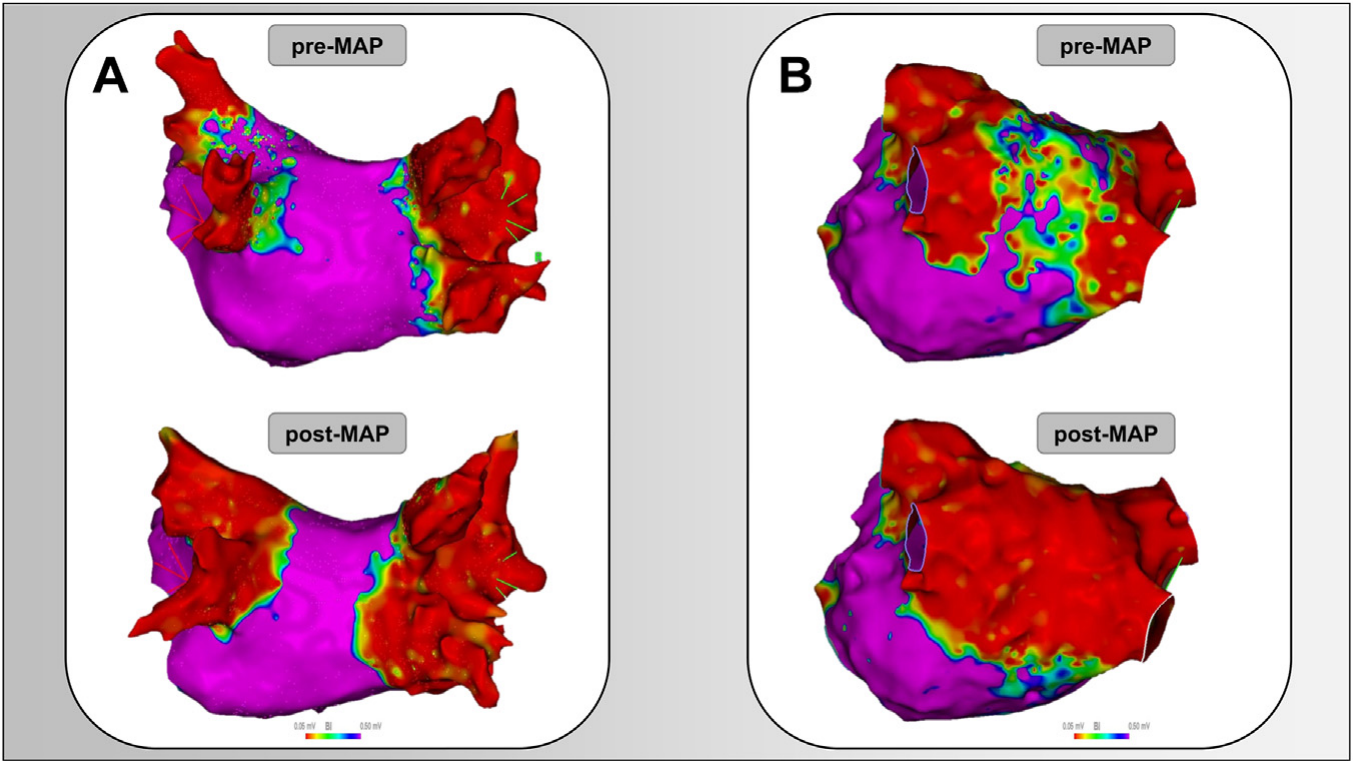
Preablation and postablation 3-dimensional electroanatomic mapping of the left atrium at repeat pulmonary vein isolation (PVI) with pulsed-field ablation for reisolation of reconnected veins and antral widening of still isolated veins after prior failed PVI with thermal ablation **(A)** and posterior wall ablation after multiple PVIs using thermal ablation with durable PVI and posterior wall scar **(B).** MAP = mapping.

At the end of the ablation procedure, the 3D EAM was repeated to verify acute PVI and/or posterior wall ablation. Figure 2 shows exemplary preablation and postablation 3D EAMs of 2 patients undergoing repeat procedure using PFA.

### Follow-up

The post-treatment plan included 7-day Holter electrocardiographic (ECG) evaluations 3, 6, and 12 months after the repeat procedure. Recurrence was defined as any ATa lasting ≥30 seconds between days 91 and 365 after the ablation after a blanking period of 90 days.

### Statistical analysis

Continuous variables are given as mean ± SD or as median [interquartile range] as appropriate. Kaplan-Meier analysis was used to estimate the rate of recurrence of atrial arrhythmias. Pairwise log-rank test were performed to assess for differences between AF phenotype groups. Comparisons between independent groups were made using the χ^2^ method for categorical variables and the Kruskal-Wallis *H* test for continuous variables. Statistical analyses were made using R 4.2.3 (R Core Team, Vienna, Austria).

## Results

### Patient characteristics

A total of 186 patients undergoing a repeat AF ablation procedure using PFA between May 2021 and December 2022 were enrolled. Of these patients, the ATa leading to repeat AF ablation using PFA was paroxysmal AF in 66 (35.5%), persistent AF in 84 (45.2%), and atypical flutter in 36 (19.3%). Median number of previous ablations was 1 (range 1–6). The ablation technology used in the previous procedure was RFA in 129 patients (69.4%), CBA in 51 (27.4%), and epicardial surgical ablation in 6 (3.2%). Baseline characteristics are summarized in Table 1.

**Table 1:** Baseline characteristics grouped by atrial tachyarrhythmia leading to this repeat PVI with PFA

	Paroxysmal AF (N = 66)	Persistent AF (N = 84)	Atrial flutter (N = 36)	*P* value∗
Age (y)	65.8 [56.6–72.3]	64.9 [57.3–70.9]	68.3 [61.4–75.7]	.256
Male sex	50 (76)	60 (71)	27 (75)	.819
BMI (kg/m^2^)	28.3 [24.9–31.9]	28.8 [25.2–33.4]	26.9 [24.4–30.7]	.098
No. of this repeat ablation				.285
First repeat procedure	46 (70)	48 (57)	22 (61)	
Multiple repeat procedure	20 (30)	36 (43)	14 (39)	
Median number of prior PVIs	1.0 [1.0–2.0]	1.0 [1.0–2.0]	1.0 [1.0–2.0]	.244
Ablation modality used for most recent PVI				.397
CBA	22 (33)	18 (21)	11 (31)	
RFA	41 (62)	64 (76)	24 (67)	
Surgery	3 (4.5)	2 (2.4)	1 (2.8)	
Type of AF at first diagnosis				**<.001**
Paroxysmal	48 (73)	20 (24)	19 (53)	
Persistent	18 (27)	64 (76)	17 (47)	
Age at first PVI (y)	61.0 [53.0–69.0]	60.5 [52.0–67.0]	65.0 [53.8–73.2]	.162
Time since AF diagnosis until first PVI (mo)	25.0 [5.2–102.0]	33.0 [14.8–70.5]	27.0 [8.2–70.8]	.911
Previous DCCV	16 (24%)	48 (57%)	12 (33%)	**<.001**
CHA_2_DS_2_-VASc score				N/A
0	17 (26)	9 (11)	2 (5.6)	
1	11 (17)	31 (37)	8 (22)	
2	25 (38)	20 (24)	13 (36)	
3	6 (9.1)	12 (14)	5 (14)	
4	6 (9.1)	8 (9.5)	5 (14)	
5	1 (1.5%)	2 (2.4%)	1 (2.8%)	
6	0 (0)	2 (2.4)	2 (5.6)	
Arterial hypertension	29 (44)	47 (56)	22 (61)	.182
Heart failure	7 (11)	22 (26)	6 (17)	**.050**
Diabetes mellitus	7 (11)	3 (3.6)	3 (8.3)	.229
Previous stroke or TIA	1 (1.5)	7 (8.3)	3 (8.3)	.146
Coronary artery disease	13 (20)	10 (12)	11 (31)	**.050**
Chronic obstructive pulmonary disease	1 (1.5)	1 (1.2)	2 (5.6)	.329
Obstructive sleep apnea syndrome	7 (11)	6 (7.1)	10 (28)	**.011**
Oral anticoagulation	49 (74)	75 (89)	34 (94)	**.008**
Left ventricular ejection fraction (%)	60.0 [55.0–65.0]	56.0 [50.0–60.0]	58.0 [45.0–60.2]	**.002**
Left atrial diameter (mm)	42.0 [38.0–46.0]	46.0 [43.0–52.0]	45.0 [40.0–50.0]	**<.001**
Left atrial volume index (mL/m^2^)	37.0 [32.0–47.0]	44.0 [37.0–62.0]	44.7 [42.2–55.5]	**.013**

Values are given as median [interquartile range] or n (%) unless otherwise indicated. Where not differently specified, baseline characteristics refer to at first PVI.

AF = atrial fibrillation; BMI = body mass index; CBA = cryoballoon ablation; DCCV = direct current cardioversion; N/A = not available; PFA = pulsed-field ablation; PVI = pulmonary vein isolation; RFA = radiofrequency ablation; TIA = transient ischemic attack.

∗ Fisher exact test, Pearson χ^2^ test, Kruskal-Wallis rank sum test. Bold *P* values are significant.

### Procedural characteristics

Comparing the procedural data grouped by the ATa leading to the repeat AF ablation using PFA, patients with atypical flutter showed longer overall procedural duration, LA dwell time, duration of ablation (from first to last PFA application), and fluoroscopy time compared to patients with persistent and paroxysmal AF (Table 2). Procedural duration was longer in case of a second or more repeat procedure (112.5 [91.8–146.8] minutes) compared to first repeat procedures (94.0 [73.5–135.2] minutes; *P* = .004).

**Table 2:** Procedural data grouped by atrial tachyarrhythmia leading to repeat procedure

	Paroxysmal AF (N = 66)	Persistent AF (N = 84)	Atrial flutter (N = 36)	*P* value∗
Procedural characteristics				
Procedural duration (min)	90.0 [70.2–112.0]	96.0 [78.8–131.2]	160.0 [135.0–217.5]	**<.001**
Fluoroscopy time (min)	16.0 [12.1–21.8]	17.6 [12.9–23.0]	22.1 [17.0–35.5]	**<.001**
Fluoroscopy dose (cGycm^2^)	503 [234–831]	472 [260–1,095]	668 [430–1,449]	.132
Left atrial dwell time (min)]	76.0 [62.0–90.0]	86.0 [71.0–109.0]	140.5 [115.5–214.2]	**<.001**
Time from first–last ablation (min)	19.5 [15.0–32.5]	24.5 [16.0–40.0]	42.0 [21.8–81.0]	**.004**
Three-dimensional electroanatomic mapping before ablation	66 (100)	83 (99)	36 (100)	>.999
No. of patients with still isolated PVs	17 (26)	39 (46)	19 (53)	**.009**
Lesion set				
Posterior wall ablation	32 (48)	65 (77)	28 (78)	**<.001**
Additional PFA targets				
Anterior/septal line	1	3	8	
Mitral isthmus/annulus	0	0	12	
SVC	1	0	0	
RA focus	1	0	1	
Additional RFA targets				
CTI	8	6	6	
SVC	4	1	3	
Posterior mitral isthmus line	2	0	10	
Anterior/Septal line	1	1	2	
RA focus	3	0	0	
LA focus	0	2	1	
Periprocedural complications				
Periprocedural cardiac tamponade	0 (0)	0 (0)	0 (0)	N/A
Phrenic nerve palsy lasting >24 h	0 (0)	0 (0)	0 (0)	N/A
Periprocedural stroke or TIA	1 (1.5)	0 (0)	0 (0)	.548
Periprocedural transient ST elevation	0 (0)	1 (1.2)	0 (0)	>.999

Values are given as median [interquartile range], n (%), or n unless otherwise indicated.

CTI = cavotricuspid isthmus; LA = left atrium; PV = pulmonary vein; RA = right atrium; SVC = superior vena cava; other abbreviations as in Table 1.

∗ Fisher exact test, Pearson χ^2^ test, Kruskal-Wallis rank-sum test. Bold *P* values are significant.

### Electrophysiological findings and subsequent lesion sets

**Figure 3:**
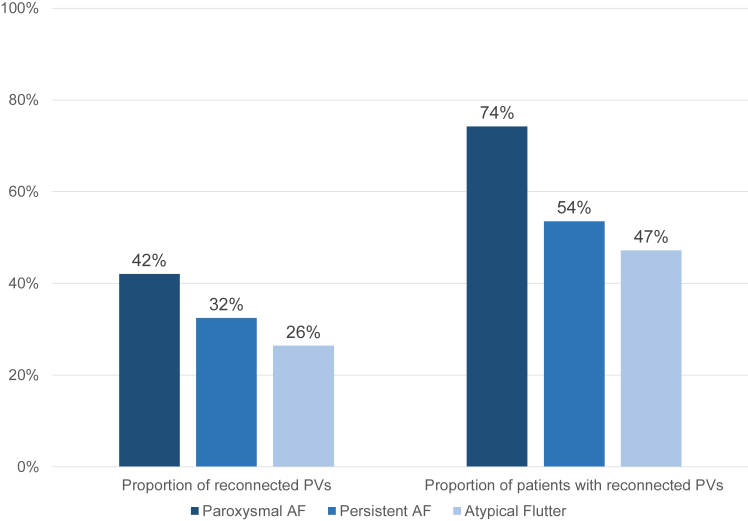
Isolation status of pulmonary veins (PVs) at repeat pulmonary vein isolation (PVI) with pulsed-field ablation for each atrial tachyarrhythmia leading to repeat PVI. AF = atrial fibrillation.

On the preablation 3D EAM, 258 of 744 PVs (35%) showed reconnections (Figure 3). On a patient level, 111 of 186 patients (60%) had at least 1 reconnected vein. This proportion was highest in the patient group with paroxysmal AF (74%), followed by patients with persistent AF (54%) and patients with atypical flutter (47%) (*P* = .009). PVI durability after 1 previous procedure was 55%, after 2 previous procedures 80%, and after >2 previous procedures 87%.

**Figure 4:**
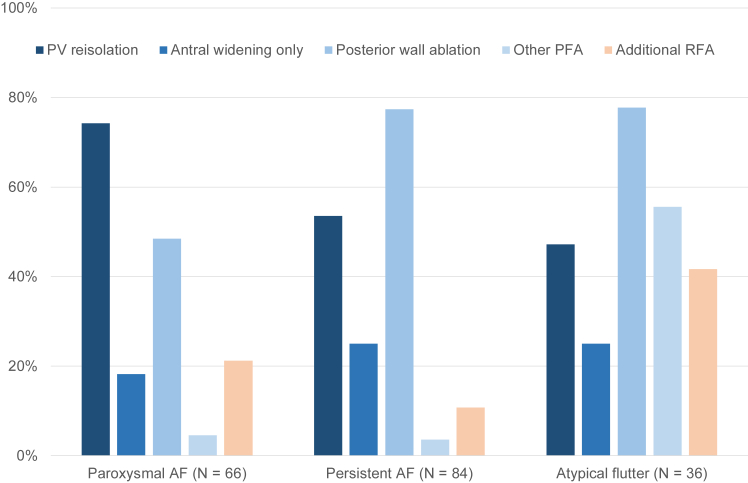
Overview of lesion set at repeat atrial fibrillation (AF) ablation for each arrhythmia type leading to repeat AF ablation. Possible other extra-pulmonary vein (PV) ablation targets using pulsed-field ablation (PFA) included septal/anterior line, mitral isthmus/annulus, superior vena cava (SVC), and focal spots of right atrium. Possible additional radiofrequency ablation (RFA) included cavotricuspid isthmus ablation, SVC, mitral isthmus/annulus, septal/anterior line, atrioventricular node, and focal spots in the right/left atrium.

An overview of the lesion sets according to type of ATa is shown in Figure 4. Reisolation of the PVs was the predominant ablation strategy in patients with paroxysmal AF. Additional antral ablations to widen the level of PV isolation were performed in 236 of 486 durably isolated veins (49%). LA posterior wall ablation was performed more often in patients with atypical flutter (78%) and persistent AF (77%) than in patients with paroxysmal AF (48%) (*P* <.001).

Additional PFA targets included the left-sided septum, mitral isthmus/annulus, superior vena cava, or posterior/lateral wall of right atrium and were used most frequently in patients with atypical flutter (56%) (Table 2). Additional RFA of the cavotricuspid isthmus (CTI), superior vena cava, mitral isthmus/annulus, anterior/septal line, or other targets was performed in 14 of 66 patients (21%) with paroxysmal AF, 9 of 84 patients (11%) with persistent AF, and 15 of 36 patients (42%) with atypical flutter (Table 2).

### Safety outcome

One patient (0.5%) suffered from a transient ischemic attack with amaurosis fugax. Magnetic resonance imaging showed no ischemic or thromboembolic intracerebral lesion. One patient (0.5%) with known ischemic coronary disease had transient ST-segment elevation in the inferior ECG leads a few minutes after additional RFA of the CTI. The ST elevation rapidly resolved. Subsequent coronary angiography showed an in-stent stenosis of the proximal right coronary artery, which was treated by balloon dilation and implantation of 2 drug-eluting stents. No patients in this study experienced persisting (>24 hours) phrenic nerve palsy, cardiac tamponade, or atrioesophageal fistula.

### Recurrences after repeat AF ablation

**Figure 5:**
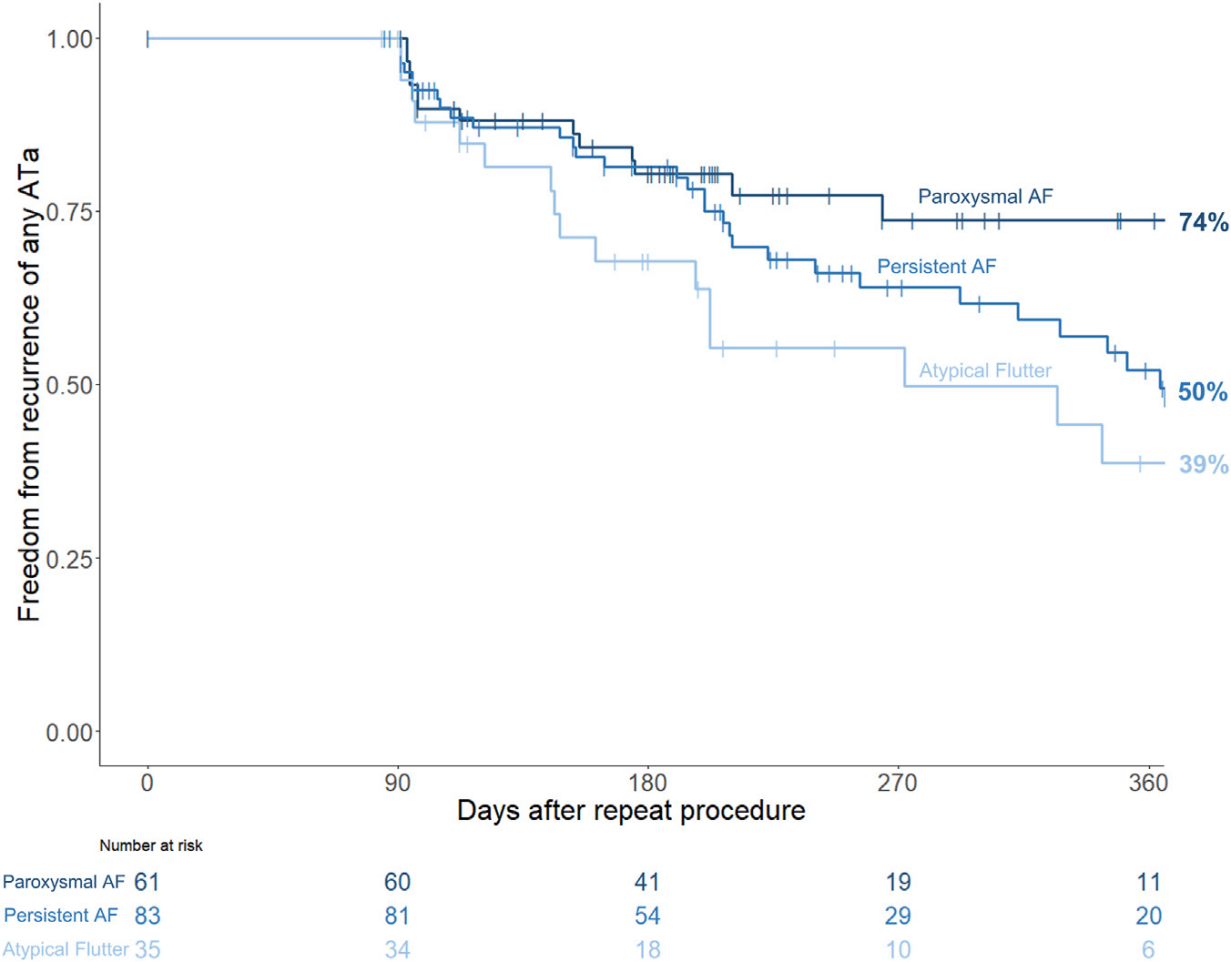
Kaplan-Meier curves of freedom from any atrial tachyarrhythmia (ATa) (atrial fibrillation [AF]/atrial flutter/atrial tachycardia) after repeat AF ablation using pulsed-field ablation (primary endpoint). Group comparison between types of ATa leading to repeat AF ablation.

Median follow-up duration after the repeat ablation procedure was 203 [129–336] days. In Kaplan-Meier analysis, freedom from recurrence of atrial arrhythmias after 6 and 12 months was 78% and 54%, respectively. In the subgroups of patients with paroxysmal AF, persistent AF, and atypical flutter, freedom from recurrence after 6 and12 months was 80% and 74%, 81% and 50% and 68% and 39%, respectively (Figure 5). Grouping freedom from recurrence by prior ablation technology, the RFA group exhibited rates of 79% at 6 months and 53% at 12 months postablation, whereas the CBA group showed percentages of 77% at 6 months and 56% at 12 months.

In an analysis of patients with persistent AF (n = 84), the recurrence rate in patients undergoing a LA posterior wall ablation was 37% (n = 24/65) as opposed to 47% in patients with no LA posterior wall ablation (n = 9/19) (*P* = .44).

In patients with paroxysmal AF, the type of ATa recurrence after the PFA repeat procedure was paroxysmal AF in 7 of 13 patients (54%), persistent AF in 2 of 13 patients (15%), and atypical flutter in 4 of 13 patients (31%). In the patient group with persistent AF, regression to paroxysmal AF occurred in 8 of 30 patients (27%), persistent AF recurred in 13 of 30 patients (43,%) and atypical flutter in 9 of 30 patients (30%). In patients with atypical flutter, it was paroxysmal AF in 4 of 16 cases (25%), persistent AF in 1 of 16 cases (6%), and atypical flutter in 11 of 16 cases (69%) (Table 3).

**Table 3:** Follow-up data grouped by ATa leading to repeat AF ablation using PFA

	Paroxysmal AF (N = 66)	Persistent AF (N = 84)	Atrial flutter (N = 36)	*P* value∗
Recurrence of any ATa during blanking period	11 (17)	12 (14)	6 (17)	.906
Recurrence of any ATa between days 91 and 365 post repeat ablation	13 (26)	30 (50)	16 (61)	.095 (log rank)
Rhythm of recurrence of any ATa				**.016**
Paroxysmal AF	7 (54)	8 (27)	4 (25)	
Persistent AF	2 (15)	13 (43)	1 (6)	
Atrial flutter	4 (31)	9 (30)	11 (69)	
Follow-up by implantable loop recorder	15 (23)	0 (0)	3 (8)	**<.001**

Values are given as n (%) unless otherwise indicated.

AF = atrial fibrillation; ATa = atrial tachyarrhythmia; PFA = pulsed-field ablation.

∗ Fisher exact test, Pearson χ^2^test; log-rank test. Bold *P* values are significant.

## Discussion

The present study is among the first to evaluate the value of PFA for repeat procedures after failed prior thermal ablation. Our results provide important insights on the use of PFA for repeat procedures, and we report 4 major findings. First, PFA was used for reisolation of reconnected veins, for antral widening, for posterior wall ablation, and for additional ablation targets, indicating a high versatility for this novel modality. Second, the use of PFA for repeat procedures was safe, and periprocedural complications were rare. Third, the combination of 3D EAM and PFA allowed for efficient workflows as indicated by relatively short procedural times. Fourth, short-term clinical outcomes after repeat procedures using PFA as the ablation modality were favorable in this initial experience.

### Procedural performance and lesion set

In routine clinical practice, patients who require repeat procedures for arrhythmia recurrences typically represent a negative selection. Consequently, in such patient populations, PV reconnections are frequently observed during a repeat procedure. Recent multicenter studies on repeat ablations following initial PVI procedures, which used thermal ablation technologies, have shown that durability rates range from 46%–64% on a per vein level, and from 10%–30% on a patient level.^12^^,^^29^

In our study, the rate of reconnections was highest in patients with paroxysmal AF (74%); hence, the PFA device was used and well suited for reisolation of the reconnected veins. In addition, it allowed widening of the antral ablation area in patients with rather ostial isolation of the PVs (Figure 2).

In patients with persistent AF (54%) or atypical flutter (47%), reconnections were less frequently observed. Therefore, extra-PV sites were more often targeted for ablation. Use of the multipolar PFA catheter, especially when used in the “flower” configuration, offers a highly efficient technique for LA posterior wall ablation.^14^ Median number of PFA applications for posterior wall ablation was 22 [18–28].

A septal or anterior line could be durably blocked by PFA in 8 of 12 cases. With regard to the use of PFA to block the posterior mitral isthmus, Davong et al^30^ reported 100% success in 45 patients using the same device. In our series, however, posterior mitral isthmus block often was only transient (10/12 cases). Consequently, additional RFA including epicardial ablation from within the coronary sinus was required to achieve a bidirectional block in some instances (n = 10).

Furthermore, in 1 patient the superior vena cava was isolated using PFA without any complications. Another case in which successful superior vena cava isolation was achieved has been described.^31^ It has to be mentioned that use of PFA for these additional ablation sites currently is off-label, and additional data, particularly with regard to safety, are needed.

Younis et al^24^ recently studied the value of PFA for redo procedures in a porcine animal study with a simulated redo environment. After performing radiofrequency PVI with intentional gaps in a first experiment, the animals underwent a second procedure 5 weeks later using PFA in the area of prior RFA to close the gaps. Repeat ablation with PFA resulted in complete isolation of the PVs and the posterior wall. In addition, they reported that PFA lesions over chronic RFA lesions were larger and deeper than RFA lesions over chronic RFA lesions. This may have a relevant impact on the outcome of repeat ablations with PFA after prior thermal ablation. Further studies in humans are needed to compare ablation technologies and strategies during repeat procedures that include redo PVI only vs those that include redo PVI plus posterior wall ablation.

### Procedural safety

In this study, none of the patients experienced a cardiac tamponade, persistent phrenic nerve palsy, or atrioesophageal fistula as a result of their repeat procedure with PFA, indicating that the procedure is safe. This excellent safety profile likely was facilitated by the use of cardiomyocyte-specific electroporation, which reduces the risk of energy-related extracardiac complications. Our findings are consistent with preclinical PFA studies, which showed similar results.^14^^,^^21^^,^^22^^,^^32^ MANIFEST-PF (Multi-National Survey on the Methods, Efficacy, and Safety on the Post-Approval Clinical Use of Pulsed Field Ablation), the first large postmarket registry to report on procedural characteristics of the PFA system, partly confirmed this observation in the first 1817 patients treated with the system.^21^ Still, they showed that transient (n = 6) and even persistent (n = 1) phrenic nerve palsy can occur after PVI with PFA.^21^ Larger patient cohorts are needed to verify these first experiences.

We present a single case of a brain magnetic resonance imaging–negative amaurosis fugax (0.54%). Of note, this complication may not be specific to PFA but rather associated with interventions in the LA in general. Previous studies have reported comparable rates of strokes/transient ischemic attacks (0.57% in EU-PORIA [EUropean Real World Outcomes with Pulsed Field AblatiOn in Patients with Symptomatic AtRIAl Fibrillation]; 0.51% in MANIFEST-PF) during PFA procedures.^21^^,^^22^

One case of transient ST-segment elevation in the inferior ECG leads right after CTI block by RFA potentially could be attributed to coronary artery spasm, which is a predescribed side effect when conducting RFA in close proximity to the right coronary artery.^33^ We considered this complication not related to PFA because of the timing of the transient ST elevation right after additional RFA. Coronary spasms are a frequent complication when using PFA for CTI block.^34^ As a result, we did not perform CTI block by PFA.

### Clinical outcomes

After the repeat procedures using PFA, the recurrence rate of any ATa after 12 months was 46%. There was no significant difference in recurrence rates between patients grouped by phenotype of recurrent ATa leading to repeat procedure. The lack of a difference might be partially explained by a relatively high proportion of patients with implantable cardiac monitors (ICMs) in the paroxysmal AF group (23%) due to previous participation in a randomized controlled study using ICM-based endpoint adjudication (COMPARE-CRYO [Comparison of PolarX and the Arctic Front Cryoballoons for PVI in Patients With Symptomatic Paroxysmal AF]; ClinicalTrials.gov Identifier: NCT04704986). The choice of monitoring strategy has great influence on the detection rate of ATa recurrences.^35^ The ICMs certainly have led to a higher detection of recurrence than would usually be observed.

A recent study indicated that there is a high occurrence of roof-dependent macroreentrant tachycardias following PVI with PFA, which may be attributed to an excessive lesion set applied to the posterior wall resulting in unintentional and incomplete posterior wall ablation during the index PVI.^36^ In our study, 13 of 43 recurrences (30%) after repeat procedures with PFA were atypical atrial flutters. Furthermore, the recent CAPLA (Catheter Ablation for Persistent Atrial Fibrillation) study by Kistler et al^37^ showed no difference in recurrence rates between persistent AF patients who underwent PVI with or without posterior wall isolation. In line with these findings, there was no statistical difference in recurrence rates between patients with persistent AF who underwent posterior wall ablation at repeat procedure and those who did not (37% vs 47%; *P* = .44). Ongoing randomized trials will clarify the value of posterior wall ablation added to PVI in patients with persistent AF (PIFPAF-PVI [Pulmonary Vein Isolation With Pulsed-Field Ablation With Versus Without Posterior Wall Ablation in Patients With Symptomatic Persistent Atrial Fibrillation]; ClinicalTrials.gov Identifier: NCT05986526).

### Study limitations

First, our study is limited by the lack of a control group. Further investigations to compare PFA and RFA for repeat ablation procedures are needed. Second, the ablation strategies during the repeat procedures were left at the discretion of the operator. Although all patients in our cohort underwent LA PFA, additional right atrial ablations were also performed in a small subset of our patients. Third, follow-up strategies included 7-day-Holter ECG in some patients and ICMs in others, with the latter being most prevalent in the group of patients with paroxysmal AF. These ICMs certainly have led to a higher detection rate of AF than usually would be observed.^35^

## Conclusion

PFA is a versatile, safe, and effective ablation option for repeat procedures after failed prior PVI using thermal ablation by means of radiofrequency energy or cryoenergy.
